# Clinicopathological characteristics and prognosis of synchronous brain metastases from non-small cell lung cancer compared with metachronous brain metastases

**DOI:** 10.3389/fonc.2024.1400792

**Published:** 2024-05-22

**Authors:** Jing Li, Xiaofang Zhang, Ye Wang, Yi Jin, Yingqiu Song, Tianlu Wang

**Affiliations:** ^1^ School of Graduate, Dalian Medical University, Dalian, Liaoning, China; ^2^ Department of Radiotherapy, Cancer Hospital of China Medical University, Liaoning Cancer Hospital and Institute, Shenyang, Liaoning, China; ^3^ School of Graduate, China Medical University, Shengyang, Liaoning, China; ^4^ Department of Breast Surgery, Liaoning Cancer Hospital and Institute, Shenyang, Liaoning, China; ^5^ Department of Radiotherapy, Cancer Hospital of China Medical University, Shenyang, Liaoning, China; ^6^ Department of Radiotherapy, Cancer Hospital of Dalian University of Technology, Shenyang, Liaoning, China; ^7^ Faculty of Medicine, Dalian University of Technology, Shenyang, Liaoning, China

**Keywords:** lung cancer, synchronous, metachronous, brain metastasis, prognosis

## Abstract

**Purpose:**

Brain metastasis (BM) from non-small cell lung cancer (NSCLC) is a serious complication severely affecting patients’ prognoses. We aimed to compare the clinicopathological features and prognosis of synchronous and metachronous BM from NSCLC.

**Methods:**

Clinical data of 461 patients with brain metastases from NSCLC who visited the Cancer Hospital of China Medical University from 2005 to 2017 were retrospectively collected. We analyzed the pathophysiological characteristics of synchronous and metachronous BM from NSCLC and survival rates of the patients. Propensity score matching analysis was used to reduce bias between groups. In addition, we used the Kaplan-Meier method for survival analysis, log-rank test to compare survival rates, and Cox proportional hazards regression model for multivariate prognosis analysis.

**Results:**

Among 461 patients with BM, the number of people who met the inclusion criteria was 400 cases, and after 1:2 propensity score matching,130 had synchronous BM and 260 had metachronous BM. The survival time was longer for metachronous BM in driver mutation-negative patients with squamous cell carcinoma than synchronous BM. Conversely, metachronous and synchronous BM with gene mutations and adenocarcinoma showed no differences in survival time. Multivariate analysis showed that metachronous BM was an independent prognostic factor for overall survival. Furthermore, the pathological type squamous cell carcinoma and Karnofsky Performance Status score <80 were independent risk factors affecting overall survival.

**Conclusion:**

BM status is an independent factor influencing patient outcome. Moreover, synchronous and metachronous BM from NSCLC differ in gene mutation profile, pathological type, and disease progression and hence require different treatments.

## Introduction

Over the last several decades, lung cancer has become one of the world’s most common cancers and a major cause of death (1–3), with non-small cell lung cancer (NSCLC), representing over 80% of cases (1, 3, 4). The most common histologic subtypes of NSCLC are lung adenocarcinoma (LUAD), squamous cell carcinoma (LUSC), and large cell carcinoma (4). Central nervous system (CNS) metastasis is a common complication of NSCLC. Data from the National Cancer Institute show that the risk of CNS metastasis in patients with LUAD, LUSC, and large cell carcinoma is 11%, 6%, and 12%, respectively (5, 6).

The most common CNS metastasis in NSCLC is brain metastasis (BM). The occurrence of BM leads to a poor prognosis and severely impacts patients’ quality of life and survival rates (6–8). The survival time of patients with NSCLC diagnosed with BM is significantly short, with an average of only 1–3 months if left untreated (6). However, in recent years, the development of different therapies, such as surgery, radiation therapy, medical interventions, and, particularly, targeted therapy, have led to better outcomes in patients with NSCLC-associated BM, improving their quality of life and prolonging their survival time.

EGFR-tyrosine kinase inhibitors (TKIs), one of the most recently introduced therapies, have demonstrated significant improvements in survival in *EGFR*-mutated advanced NSCLC (9–12), becoming the therapeutic choice for patients with advanced NSCLC and *EGFR*-sensitive mutations.

Unfortunately, due to the blood-brain barrier (BBB), most of the available drugs cannot effectively enter brain tissue, resulting in poor therapeutic efficacy. As a consequence, the 5-year survival rate of patients with BM remains low (13). BM is classified according to the time elapsed between its occurrence and lung cancer diagnosis. However, in various literatures, the criteria for distinguishing the timing of synchronous and metachronous brain metastases vary. In this paper, we consider that synchronous BM is detected within 2 months after the diagnosis of NSCLC, while metachronous BM is detected after 2 months following the NSCLC diagnosis (14, 15). The incidence rate of NSCLC-related BM is 10% (5, 16, 17) and increases with disease progression, reaching up to 40–50% in cases of advanced disease.

The factors influencing prognosis and treatment choices for NSCLC-associated BM remain unclear. In this study, we performed a retrospective analysis involving 400 patients with NSCLC, comparing the pathophysiological characteristics and survival rates between those with metachronous BM and those with synchronous BM. We aimed to determine the factors affecting NSCLC-related BM progression and explore whether patients can benefit from personalized treatment plans according to the time of BM onset.

## Materials and methods

### Study population

We collected data from a unicentral retrospective cohort of 461 patients with NSCLC-associated BM, who visited the Cancer Hospital of China Medical University between January 2005 and December 2017. The inclusion criteria encompassed (1) age ≥18 years; (2) a confirmed pathological diagnosis of NSCLC through tracheoscopy, lung puncture biopsy, metastasis biopsy, or surgical biopsy; (3) a pathological diagnosis of LUSC or LUAD; (4) NSCLC-induced BM confirmed by imaging and/or pathology, such as head-enhanced magnetic resonance imaging (MRI); (5) access to complete clinical data and follow-up information; (6) the absence of other malignancies. All enrolled patients who did not have an endpoint event (death) were followed up through outpatient visits, inpatient care, or telephone follow-up for at least 1 year.

### Data collection

Various pathological variables in patients at the time of their BM diagnosis were documented, including age (< 65 vs. ≥ 65), gender (male vs. female), pathological type (LUSC vs. LUAD), general health status score assessed by the Karnofsky Performance Status (KPS < 80 vs. ≥ 80), synchronous BM occurrence (yes/no), number of intracranial metastases (single vs. multiple), number of extra-cranial organs affected (1–2 vs. > 2), radiotherapy (yes/no), surgery (yes/no), chemotherapy (yes/no), EGFR gene mutation(yes/no), and PET-CT (yes/no). Overall survival is defined as the date from the date of BM diagnosis to the date of patient death or last follow-up visit.

### Statistical methods

IBM SPSS 26.0 software was used to create patient groups with synchronous versus metachronous BM originating from lung cancer through a 1:2 propensity score matching (PSM) approach. This was done to reduce selection bias and account for potential confounding variables. Univariate and multivariate Cox proportional hazards regression analyses, for both the raw and the propensity score-matched dataset, were used to assess survival risk factors. Variables with *p*-values < 0.1 in the univariate analysis were included in the multivariate analysis. Results were expressed as hazard ratio (HR) and 95% confidence interval (CI). Additionally, a univariate Cox proportional risk regression analysis was used to assess the effect of gene mutation and pathological type on survival stratification in patients with synchronous versus metachronous BM. Kaplan–Meier analysis and a two-sided log-rank test were used to assess the effect of the type of lung cancer and the presence of driver mutations on survival outcomes. A chi-square test was performed using R 4.2.1 to analyze the baseline characteristics before and after PSM. A Cox proportional risk regression model was used for assessing prognostic correlations, survival curve plotting, and survival data analysis. All hypothesis tests were two-sided, and results were considered significant when *p*-values were less than 0.05.

## Result

### Patient characteristics

**Figure 1:**
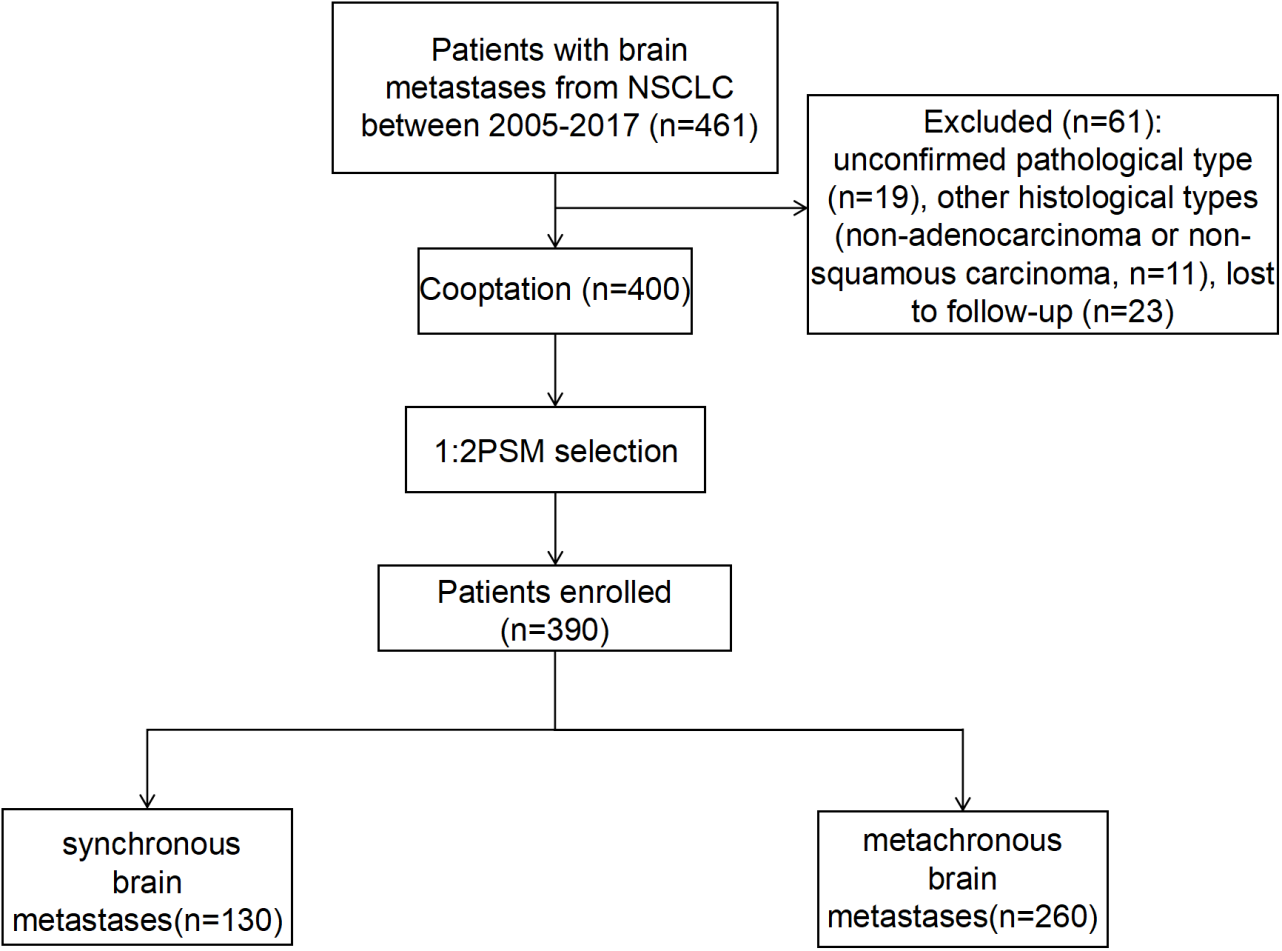
The flow chart for the selection of the study population. NSCLC, non-small cell lung cancer.

A total of 461 patients who were initially diagnosed with BM from NSCLC and admitted to the Cancer Hospital of China Medical University from March 2005 to December 2017 were included in this study. The selection process resulted in the exclusion of 61 patients for the following reasons: 19 patients due to an unclear diagnosis of pathological types, 11 patients with pathological types other than lung adenocarcinoma and squamous lung cancer, 23 patients due to missed visits, and 8 patients with incomplete clinical information. Ultimately, 400 patients were included in this study. Among them, 130 patients had synchronous BM and 270 patients had metachronous BM. On this basis, a 1:2 PSM was performed, resulting in 130 patients with synchronous metastases and 260 patients with metachronous metastases, achieving a balanced distribution between the groups (**Figure 1**).

Clinical features of the 400 patients are presented in **Table 1**. There was no significant stats discrepancy between the two groups of patients with synchronous versus metachronous brain metastases from lung cancer before PSM in terms of age at diagnosis of brain metastases [years], gender, KPS score, type of pathology, number of metastases, number of affected extracranial organs, brain surgery, brain radiotherapy, EGFR genetic mutations, and PET-CT (*p* > 0.05). There were between-group differences in whether chemotherapy was administered (*p* < 0.05), and further equalization of baseline characteristics was required. the distribution of baseline characteristics was well balanced between the two groups of patients with synchronous and metachronous brain metastases after PSM, and no statistically significant difference was observed between the two groups (*p* > 0.05).

**Table 1:** Baseline characteristics of patients with synchronous versus metachronous brain metastases before and after PSM.

Characteristic	Before PSM			After PSM		
	synchronous brain n=130	metachronous brain n=270	*p*	Synchronous brain n=130	metachronous brain n=260	*p*
Age						
<65	74 (18.5%)	157 (39.2%)	0.816	74 (19%)	150(38.5%)	0.885
>=65	56 (14%)	113 (28.2%)		56 (14.4%)	110 (28.2%)	
Gender						
Female	64 (16%)	132 (33%)	0.949	64 (16.4%)	127 (32.6%)	0.934
Male	66 (16.5%)	138 (34.5%)		66 (16.9%)	133 (34.1%)	
Pathological type						
Squamous carcinoma	19 (4.8%)	38 (9.5%)	0.885	19 (4.9%)	37 (9.5%)	0.919
Adenocarcinoma	111 (27.8%)	232 (58%)		111 (28.5%)	223 (57.2%)	
KPS						
<80	83 (20.8%)	171 (42.8%)	0.921	83 (21.3%)	166 (42.6%)	1.000
>=80	47 (11.8%)	99 (24.8%)		47 (12.1%)	94 (24.1%)	
No. of intracranial metastases						
Single shot	83 (20.8%)	175 (43.8%)	0.850	83 (21.3%)	168 (43.1%)	0.881
Multi-incidence	47 (11.8%)	95 (23.8%)		47 (12.1%)	92 (23.6%)	
Number of affected extra-cranial organs						
1-2	115 (28.7%)	223 (55.8%)	0.129	115 (29.5%)	214 (54.9%)	0.115
>2	15 (3.8%)	47 (11.8%)		15 (3.8%)	46 (11.8%)	
radiotherapy						
No	42 (10.5%)	65 (16.2%)	0.081	42 (10.8%)	62 (15.9%)	0.075
Yes	88 (22%)	205 (51.2%)		88 (22.6%)	198 (50.8%)	
Surgery						
NO	123 (30.8%)	256 (64%)	0.933	123 (31.5%)	247 (63.3%)	0.871
YES	7 (1.8%)	14 (3.5%)		7 (1.8%)	13 (3.3%)	
Chemotherapy						
NO	49 (12.2%)	132 (33%)	0.035	49 (12.6%)	124 (31.8%)	0.061
YES	81 (20.2%)	138 (34.5%)		81 (20.8%)	136 (34.9%)	
Genetic mutations						
NO	99 (24.8%)	210 (52.5%)	0.717	99 (19%)	202 (51.8%)	0.733
YES	31 (7.8%)	60 (15%)		31 (7.9%)	58 (14.9%)	
PET-CT						
NO	109 (27.3%)	238 (59.5%)	0.235	109 (27.9%)	229 (58.7%)	0.247
YES	21 (5.2%)	32 (8%)		21 (5.4%)	31 (7.9%)	

A total of 89 cases were positive for gene mutations after PSM. PSM, propensity score matching

### Impact of metachronous brain metastasis on overall survival rate

Univariate analyses were used to analyze the influence of different covariates on OS. In the univariate analysis conducted before PSM, synchronous BM (HR: 1.242 [95% CI: 1.003–1.539], *p* = 0.047), squamous carcinoma (HR: 1.522 [95% CI: 1.145–2.024], *p* = 0.004), and KPS score < 80 (HR: 1.264 [95% CI: 1.024–1.558, *p =* 0.029) were identified as risk factors affecting OS of BM in patients with NSCLC. On the other hand, female sex (HR: 0.725 [95% CI: 0.592– 0.889], *p* = 0.002) and EGFR gene positivity (HR: 0.656 [95% CI: 0.513–0.839], *p* < 0.001) were identified as significant protective factors associated with longer OS. Multifactor analysis was performed for elements with *p-*values less than 0.1 in the univariate analysis (**Table 2**), depicting synchronous BM (HR: 1.335 [95% CI: 1.076–1.657], *p* = 0.009), pathological type of squamous carcinoma (HR: 1.361 [95% CI: 1.018–1.820], *p* = 0.037), KPS score < 80 (HR: 1.392 [95% CI: 1.124–1.724], *p* = 0.002) as risk factors affecting OS of BM in patients with NSCLC.

**Table 2:** Univariate and Multivariate analysis of the effect of NSCLC brain metastasis on OS (before PSM).

Characteristics	Total(N)	Univariate analysis		Multivariate analysis	
		Hazard ratio (95% CI)	*P* value	Hazard ratio (95% CI)	*P* value
Age of diagnosis of brain metastases	400		0.291		
<65	231	Reference			
≥65	169	1.116 (0.911 - 1.366)	0.289		
Gender	400		**0.002**		
Male	204	Reference		Reference	
Female	196	0.725 (0.592 - 0.889)	**0.002**	0.825 (0.666 - 1.023)	0.079
Brain metastases status	400		0.050		
metachronous	270	Reference		Reference	
synchronous	130	1.242 (1.003 - 1.539)	**0.047**	1.335 (1.076 - 1.657)	**0.009**
Pathological type	400		**0.006**		
Adenocarcinoma	343	Reference		Reference	
squamous carcinoma	57	1.522 (1.145 - 2.024)	**0.004**	1.361 (1.018 - 1.820)	**0.037**
KPS	400		**0.027**		
≥80	146	Reference		Reference	
<80	254	1.264 (1.024 - 1.558)	**0.029**	1.392 (1.124 - 1.724)	**0.002**
No. of intracranial metastases	400		0.782		
Multiple	142	Reference			
Single	258	1.030 (0.835 - 1.270)	0.783		
Number of affected extra-cranial organs	400		0.573		
1-2	338	Reference			
>2	62	1.084 (0.822 - 1.428)	0.569		
radiotherapy	400		0.807		
YES	293	Reference			
NO	107	0.972 (0.773 - 1.222)	0.807		
Surgery	400		0.332		
YES	21	Reference			
NO	379	1.244 (0.788 - 1.964)	0.348		
Chemotherapy	400		0.336		
NO	181	Reference			
YES	219	0.905 (0.740 - 1.108)	0.335		
Genetic mutations	400		**<0.001**		
NO	309	Reference		Reference	
YES	91	0.656 (0.513 - 0.839)	**<0.001**	0.914 (0.697 - 1.198)	0.514
PET-CT	400		0.093		
NO	347	Reference			
YES	53	0.779 (0.576 - 1.052)	0.103		

PSM, propensity score matching; NSCLC, non-small cell lung cancer; OS, overall survival.Bold values means results were considered significant when p-values were less than 0.05.

**Figure 2:**
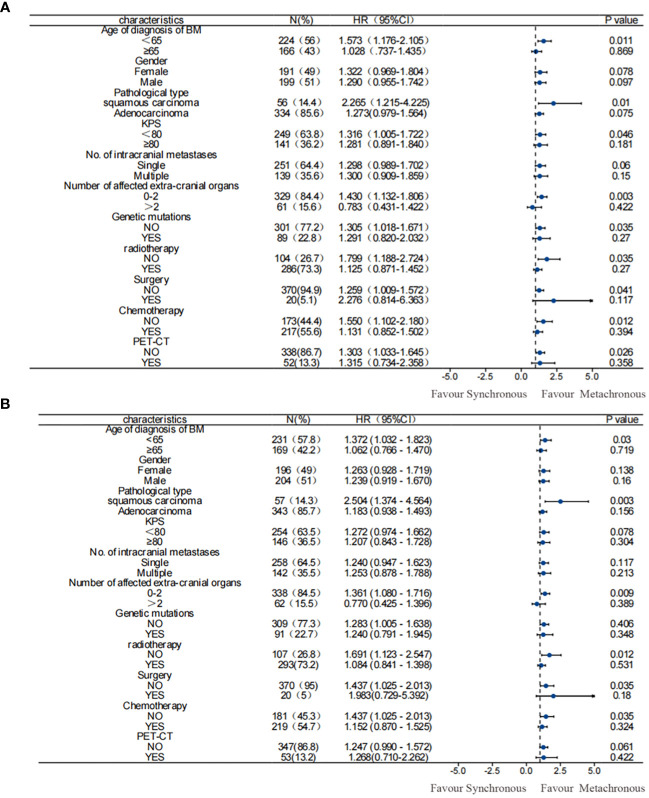
Subgroup analysis of OS among patients. **(A)** before PSM; **(B)** after PSM. Results before and after PSM suggested that metachronous BM was associated with significantly longer OS. PSM, propensity score matching; OS, overall survival.

The univariate and multivariate analysis results for 390 patients with NSCLC-associated BM after PSM were generally consistent with those before PSM. In the univariate analysis following PSM: a male sex, a pathological type of squamous carcinoma, KPS score < 80, and EGFR gene negativity were identified as risk factors for OS in patients with NSCLC exhibiting BM. On the other hand, metachronous BM (HR: 0.771 [95% CI: 0.622–0.957], *p* = 0.018) emerged as a protective factor for OS. The multifactor analysis was performed on factors with *p*-values less than 0.1 in the results after univariate analysis (**Table 3**), identified metachronous BM (HR: 0.715 [95% CI: 0.575–0.889], *p* = 0.002) as an independent protective factor for OS; whereas the pathological type of squamous carcinoma and KPS score < 80 were identified as independent risk factors for OS in NSCLC-associated BM. Moreover, subgroup analyses according to patients’ clinical characteristics confirmed that metachronous BM was associated with a significantly longer OS in all subgroups (**Figure 2**).

**Table 3:** Univariate and Multivariate analysis of the effect of NSCLC brain metastasis on OS (after PSM).

Characteristics	Total(N)	Univariate analysis		Multivariate analysis	
		Hazard ratio (95% CI)	*P* value	Hazard (95% CI)	*P* value
Age of diagnosis of brain metastases	390		0.232		
<65	224	Reference			
≥65	166	1.134 (0.923 - 1.392)	0.231		
Gender	390		**0.002**		
Female	191	Reference		Reference	
Male	199	1.392 (1.133 - 1.711)	**0.002**	1.221 (0.983 -1.518)	0.072
brain metastases status	390		**0.020**		
synchronous	130	Reference		Reference	
metachronous	260	0.771 (0.622 - 0.957)	**0.018**	0.715 (0.575- 0.889)	**0.002**
Pathological type	390		**0.004**		
Adenocarcinoma	334	Reference		Reference	
squamous carcinoma	56	1.552 (1.164 - 2.069)	**0.003**	1.397 (1.041 -1.873)	**0.026**
KPS	390		**0.017**		
≥80	141	Reference		Reference	
<80	249	1.292 (1.044 - 1.598)	**0.019**	1.430 (1.151 -1.777)	**0.001**
No. of intracranial metastases	390		0.828		
Multiple	139	Reference			
Single	251	1.024 (0.828 - 1.265)	0.828		
Number of extracranial organs affected	390		0.510		
1-2	329	Reference			
>2	61	1.099 (0.832 - 1.453)	0.505		
radiotherapy	390		0.769		
YES	286	Reference			
NO	104	0.966 (0.765 - 1.219)	0.770		
surgery	390		0.278		
NO	370	Reference			
YES	20	0.779 (0.488 - 1.244)	0.296		
Chemotherapy	390		0.543		
YES	217	Reference			
NO	173	1.066 (0.868 - 1.309)	0.542		
Genetic mutations	390		**<0.001**		
YES	89	Reference		Reference	
NO	301	1.532 (1.194 - 1.966)	**<0.001**	1.094 (0.831 - 1.441)	0.520
PET-CT	390		0.098		
YES	52	Reference			
NO	338	1.283 (0.947 - 1.739)	0.108		

PSM, propensity score matching; NSCLC, non-small cell lung cancer.Bold values means results were considered significant when p-values were less than 0.05.

**Figure 3:**
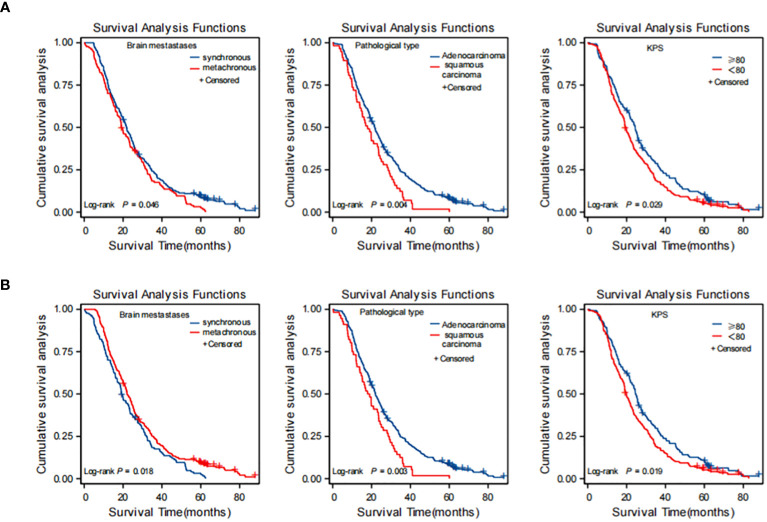
Kaplan-Meier curves for each independent factor affecting overall survival (OS) in NSCLC brain metastasis (BM) patients. **(A)** before PSM; **(B)** after PSM. **(A)** before PSM: synchronous brain metastases, squamous carcinoma, KPS score <80 were risk factors affecting OS of brain metastasis in NSCLC. **(B)** after PSM: metachronous brain metastases was an independent protective factor for OS; squamous carcinoma and KPS score <80 were independent risk factors for OS in brain metastases from NSCLC. PSM, propensity score matching; NSCLC, non-small cell lung cancer.

The Kaplan–Meier curves of OS in patients with BM from lung cancer according to the results of multivariate analysis conducted before and after PSM, under each independent factor, showed that metachronous BM is associated with a significantly longer OS (**Figure 3**).

### Pathology type and EGFR genes of synchronous and metachronous BM

**Figure 4:**
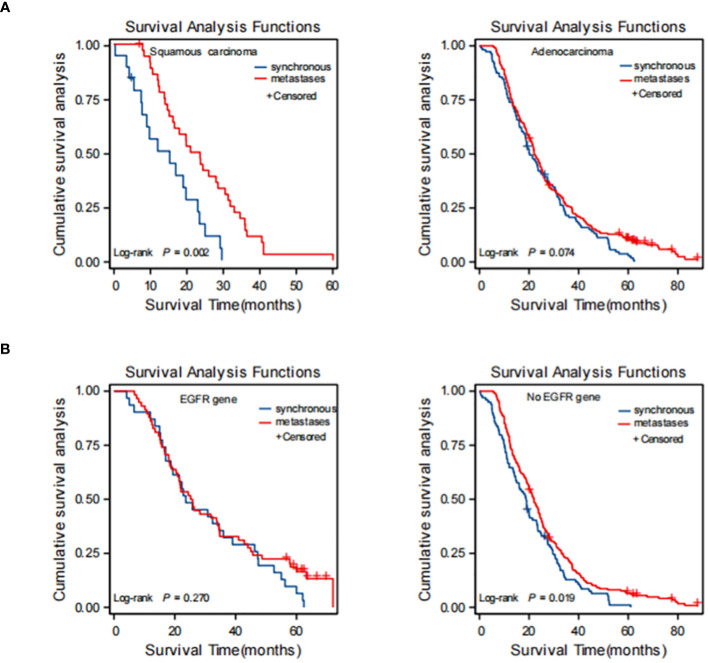
Kaplan-Meier curve of synchronous BM and metachronous BM based on the results of stratification analysis: type of patholog **(A)** and driver genes **(B)**. Type of pathologypatients with metachronous brain metastases with pathological type of squamous carcinoma had longer OS than synchronous brain metastases. Driver genes: patients with metachronous brain metastases with negative EGFR genes had longer OS than synchronous brain metastases. BM, brain metastasis; OS, overall survival; EGFR, epidermal growth factor receptor.

To further evaluate the prognostic impact of synchronous versus metachronous BM on patients with NSCLC, we analyzed the relationship between the presence or absence of synchronous BM and the pathological type and EGFR driver genes. The impact on OS in patients with synchronous and metachronous BM showed no significant difference when considering the pathological type of adenocarcinoma (*p* = 0.075). However, patients with metachronous BM of squamous carcinoma had longer OS than those with synchronous BM (*p* = 0.01) (**Table 4** and **Figure 4A**). In terms of EGFR genes, there was no difference in the effect of synchronous BM with positive EGFR genes versus metachronous BM on OS (*p* = 0.270). However, metachronous BM with negative EGFR genes had longer OS compared to those with synchronous BM (*p* = 0.044) (**Table 4** and **Figure 4B**).

**Table 4:** Stratified analysis of pathological types and driver genes before and after PSM.

Characteristics	Variable	Before PSM		After PSM	
		Hazard ratio (95% CI)	*P* value	Hazard ratio (95% CI)	P value
Squamous carcinoma	synchronous brain metastases	REF		REF	
	metachronous brain metastases	2.504 (1.374-4.564)	0.003	2.271 (1.217-4.235)	**0.010**
Adenocarcinoma	synchronous brain metastases	REF		REF	
	metachronous brain metastases	1.183 (0.938-1.493)	0.156	1.237 (0.979-1.564)	0.075
EGFR Genetic mutations	synchronous brain metastases	REF		REF	
	Metachronous brain metastases	1.240 (0.791-1.945)	0.348	1.291 (0.820-2.032)	0.270
No EGFR genetic mutation	synchronous brain metastases	REF		REF	
	Metachronous brain metastases	1.283 (1.005-1.638)	**0.046**	1.291 (1.007-1.656)	**0.044**

PSM, propensity score matching; EGFR, epidermal growth factor receptor.Bold values means results were considered significant when p-values were less than 0.05.

### Distribution characteristics of patients with BM

**Figure 5:**
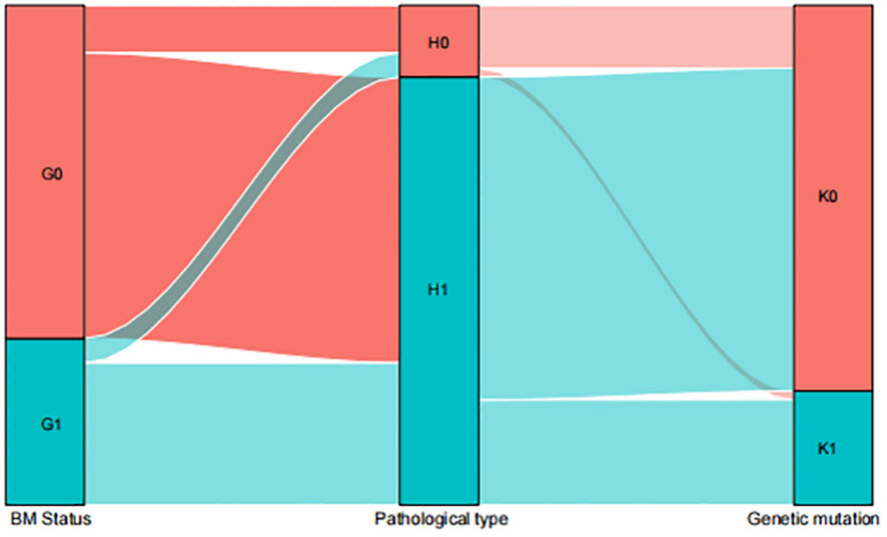
Sankey map among the influencing factors. G0=metachronous G1=synchronous; H0=LUAD H1=LUSC; K0=NO EGFR genetic mutations K1=EGFR genetic mutations.

The distribution characteristics of patients with BM are illustrated in Sankey plots. These plots visualize the relationship between BM status, pathological type, and EGFR gene mutation. The analysis indicates that the pathological type of synchronous BM and metachronous BM is mostly LUSC and most of the LUSC do not exhibit gene mutations (**Figure 5**).

### Patients with adenocarcinoma with positive EGFR had longer OS at 1,2, and 3 years

**Figure 6:**
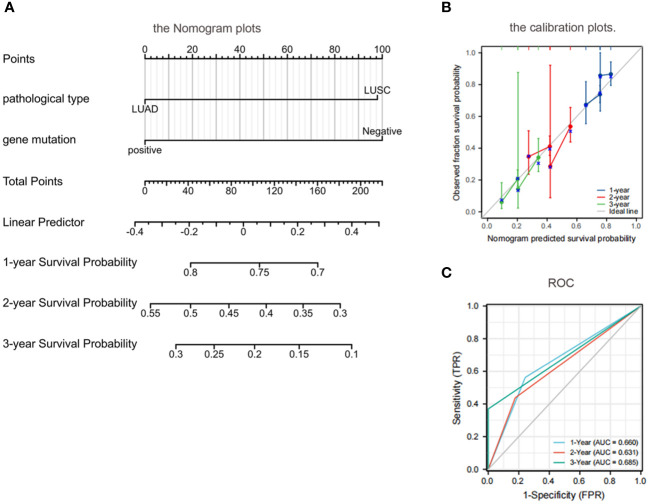
Prognostic modeling and ROC curves by nomogram. **(A)** Nomogram plots: patients with adenocarcinoma with a positive EGFR gene had higher OS at 1 year, 2 years, and 3 years than those with a negative squamous cell carcinoma with EGFR; **(B)** Calibration plots further validate the predictive performance of the nomogram; **(C)** ROC: the AUC was between 0.5 and 1.0, indicating a decent level of discriminative ability for the nomogram. OS, overall survival; EGFR, epidermal growth factor receptor; ROC, Receiver operating characteristic; AUC, area under the curve.

According to the results of univariate analysis and multivariate analysis using the COX proportional hazards regression model, we incorporated pathological types and EGFR genes into the model. Subsequently, Nomogram plots of the 1-year, 2-year, and 3-year survival probabilities of lung cancer with BM were generated (**Figure 6**). The Nomogram plots enable the assignment of scores for each variable by drawing an upward vertical line along the score axis for each assigned variable. These scores are then accumulated to determine the prognostic score for each variable. According to the prognosis score of each patient, a vertical line along the probability axis is drawn downward, to obtain the corresponding 1-year, 2-year, and 3-year survival probability values.

The calibration of the nomogram was assessed through calibration plots. Nomogram predicted and actual 1-year, 2-year, and 3-year OS rates were plotted and compared to further validate the predictive performance of the nomogram (**Figure 6**).

The discriminative ability of the nomogram to predict 1-year, 2-year, and 3-year OS was assessed using the area under the curve (AUC) of the receiver operating characteristic curve (ROC). The C indices of 1-year, 2-year, and 3-year predicted survival were 0.660, 0.631, and 0.685, respectively. The AUC was between 0.5 and 1.0, indicating a decent level of discriminative ability for the nomogram, with 0.5 representing a random outcome (**Figure 6**).

## Discussion

The fundamental reason for the difference in treatment between synchronous and metachronous BM in NSCLC is the timing of BM. Synchronous BM refers to the detection of BM within 2 months after diagnosis of NSCLC.At this time, targeted BM treatment should be carried out in addition to systemic treatment. Metachronous BM refers to BM detected 2 months after diagnosis of NSCLC.Active treatment should be given to the primary focus of the lung cancer first, and once BM occurs, treatment should be directed towards it.

Some previous studies have also shown that the prognosis of patients with synchronous BM is worse than that of patients with metachronous BM (18, 19). In other studies, there was no difference in median OS between patients with synchronous metastases and those with metachronous metastases. Nevertheless, the main limitation of these studies was the small number of patients included and unconvincing conclusions (20, 21). In addition, patients were treated mainly with radiotherapy and chemotherapy, in which there may be a time shift in analysis. In this study, we obtained data from 400 patients (including 130 patients with synchronous and 270 with metachronous BM). The results showed that metachronous BM was a protective factor for OS (*p* = 0.002).

Considering the heterogeneity of pathological factors, we excluded large cell lung cancer and other subtypes of lung cancer. We only collected data from the two most common pathological types of NSCLC: LUAD and LUSC. Our results showed that the pathological type of NSCLC correlated with BM status. Furthermore, the pathological type was an independent prognostic factor affecting OS. Additionally, we found significant differences in prognosis between LUAD and LUSC: patients with LUAD exhibited significantly longer OS compared to those with LUSC, a pattern consistent with previous studies (22–24).

To address potential selection bias between driver mutation-negative and mutation-positive LUAD and LUSC cases, we performed a stratified analysis. Within the dataset of patients with BM from lung cancer, patients underwent mutation gene testing using next-generation sequencing (NGS) technology, and 89 patients were found to be positive for driver gene mutations, of which 81 patients exhibited EGFR gene mutations. There were 31 simultaneous BM (all EGFR mutations) and 58 metachronous BM. Among these, 50 patients had EGFR mutations and the remaining 8 exhibited ALK mutations. Our results showed a difference in survival rates between metachronous and synchronous BM in EGFR mutation-negative patients with LUSC, whereas there was no difference in survival between metachronous and synchronous BM in EGFR mutation-positive patients with LUAD. Three generations of targeted drugs for EGFR mutations have emerged; FLAURA research shows that the prognosis of BM treated with third-generation targeted drugs is better and the progression rate is lower (25). Our findings also suggest that patients with BM exhibiting positive EGFR gene mutation experience better treatment outcomes when subjected to targeted therapy. On the other hand, for patients with EGFR mutation-negative LUSC, treatment options are limited, and the disease progresses faster. Therefore, in the context of EGFR gene mutations, synchronous BM and metachronous BM necessitate different treatment approaches, with targeted therapy offering prolonged survival and good prognosis among patients with positive EGFR gene mutations (9–12).

Currently, driver mutation-negative patients have more treatment options, owing to the rise of immunotherapy. Immune checkpoint inhibitors (ICIs) have revolutionized the treatment of patients with advanced driver gene-negative NSCLC, increasing the 5-year survival rate from 5% (26) during the chemotherapy era to 13.4–23.2% (27). This may be related to programmed death-ligand 1 (PD-L1) expression, with ICIs providing more pronounced benefit to patients with PD-L1 expression (tumor proportion score ≥ 50%). Still, some patients with negative PD-L1 expression still have achieved good progression-free survival with first-line immunotherapy (28). However, due to the complexity of the immunotherapeutic mechanism and the uncertainty of predicting the efficacy of immunotherapy by PD-L1 expression status alone, further studies are needed to determine which patients are more likely to benefit from ICIs. However, patients with EGFR mutations have certainly limited benefit from first-line immunotherapy (29).

One of the limitations of our study is that we included data from cases recorded before the formal approval of immunotherapy, resulting in the absence of information regarding immunotherapy. In the future, we will be expanding the sample size will enable us to explore the efficacy of immunotherapy in driver mutation-negative patients and LUSC. Indeed, disease progression differs among patients with different types of lung cancer-related BM. Consequently, tailoring individualized treatment plans based on patient characteristics is of paramount importance.

## Data availability statement

The original contributions presented in the study are included in the article/supplementary material. Further inquiries can be directed to the corresponding authors.

## Ethics statement

The studies involving humans were approved by Medical Ethics Committee of Liaoning Cancer Hospital and the number is 2020X0102. The studies were conducted in accordance with the local legislation and institutional requirements. The participants provided their written informed consent to participate in this study.

## Author contributions

JL: Writing – review & editing, Writing – original draft. XZ: Writing – review & editing. YW: Writing – review & editing. YJ: Writing – review & editing, Funding acquisition. YS: Writing – review & editing, Funding acquisition. TW: Writing – review & editing.
